# Design, Repeatability, and Comparison to Literature Data of a New Noninvasive Device Called “Rotameter” to Measure Rotational Knee Laxity

**DOI:** 10.1155/2015/439095

**Published:** 2015-06-28

**Authors:** Simon Neumann, Stefan Maas, Danièle Waldmann, Pierre-Louis Ricci, Arno Zürbes, Pierre-Jean Arnoux, Jens Kelm

**Affiliations:** ^1^Research Unit in Engineering Science, University of Luxembourg, 1359 Kirchberg, Luxembourg; ^2^Fachhochschule Bingen, 55411 Bingen am Rhein, Germany; ^3^Laboratoire de Biomécanique Appliquée, Université de la Méditerranée, 13916 Marseille, France; ^4^Chirurgisch-Orthopädisches MVZ, 66557 Illingen, Saarland, Germany; ^5^Klinik für Orthopädie und Orthopädische Chirurgie, Universitätsklinikum des Saarlandes, 66424 Homburg, Saarland, Germany

## Abstract

The present paper deals with the design, the repeatability, and the comparison to literature data of a new measuring device called “Rotameter” to characterize the rotational knee laxity or the tibia-femoral rotation (TFR). The initial prototype P1 of the Rotameter is shortly introduced and then modified according to trials carried out on a prosthetic leg and on five healthy volunteers, leading therefore to an improved prototype P2. A comparison of results obtained from P1 and P2 with the same male subject shows the enhancements of P2. Intertester and intratester repeatability of this new device were shown and it was observed that rotational laxities of left and right knees are the same for a healthy subject. Moreover, a literature review showed that measurements with P2 presented lower TFR values than other noninvasive devices. The measured TFR versus torque characteristic was quite similar to other invasive devices, which are more difficult to use and harmful to the patient. Hence, our prototype P2 proved to be an easy-to-use and suitable device for quantifying rotational knee laxity. A forthcoming study will validate the Rotameter thanks to an approach based on computed tomography in order to evaluate its precision.

## 1. Introduction

The knee is a voluminous and complex human joint [1, 2]. Located between the distal end of the femur and the proximal end of the tibia, it provides extension, flexion, and some rotations. It is the joint that bears three times the body weight during slow walking [3, 4]. A number of ligaments help controlling the movements and supply stability. The Anterior Cruciate Ligament (ACL) and the Posterior Cruciate Ligament (PCL) provide stability in the sagittal plane and during rotation.

After a violent twisting, for instance, in sport accidents, the ACL can tear. These ACL ruptures influence the stability of the knee by increasing its rotational laxity [5] and might damage its integrity [6]. Although reconstructive surgery permits sufficient restoration of the ligament function in sagittal plane [7], it remains limited to the restoration of rotational stability [8, 9].

There was a lot of research in the area of anteroposterior translation of the knee to detect ligament dysfunction but very little has been done concerning the rotation. Therefore, the “Rotameter” was developed to provide a noninvasive measurement of knee rotation versus applied torque to assist clinicians in diagnosing knee injuries and to assess in an objective manner reconstructive ligamentous surgery. It may be checked whether the treatment restoring the stability in the sagittal plane was effective. Furthermore, very often only one knee is injured and hence both sides can be compared.

The diagnosis of an abnormality in the knee by passive testing is a critical step in a clinical checkup. The Lachman test, the anterior and posterior drawer tests, and the pivot shift test enable the diagnosis of a failure in the ACL or the PCL. According to Malanga et al. [10], the results of these tests are directly influenced by the experience of the clinician and are limited to the diagnosis of anteroposterior translation, which is not representative for the rotational knee laxity.

Nowadays, there are some device-based approaches to measure anteroposterior translation or rotational laxity in order to promote consistency among diagnoses. Much research work is ongoing in the area of anteroposterior translation. This helped to develop devices such as the KT-1000/2000 arthrometer, the Genucom Knee Analysis System, and the Rolimeter. Cannon and Dilworth [11] and Draganich et al. [12] got familiar with these devices to measure anteroposterior laxity. Nevertheless, they recommend the use of these devices in conjunction with passive testing for effective diagnosis. Furthermore, apart from the Genucom Knee Analysis System, they provide no information about rotational laxity.

The measurement of rotational laxity is complex because of the knee's kinematics. Until now, it has been difficult to give a precise value for this rotation, though imaging medical techniques have revolutionized measurement and provide much smaller angular values compared to the existing noninvasive methods [13–22]. Additionally, noninvasive processes involving electromagnetic trackers make it possible to measure small rotational angles [23, 24]. However, they are influenced by soft tissue artefacts and the interference between metals and magnetic sources can alter the results. Some invasive methods that require the use of bone pins [23] are complex, expensive, and unsafe for patients.

**Figure 1:**
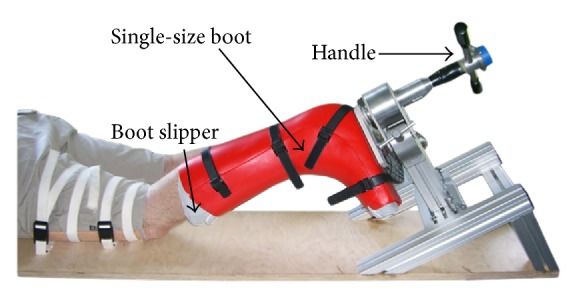
First prototype P1 of the Rotameter.

In vivo studies enable the assessment of the rotational angle of the tibia with respect to the femur [12, 25–31]. Similarly, the Rotameter has been developed as a noninvasive measurement device for the TFR versus the applied torque, which can provide objective and repeatable information. The first prototype P1 in Figure 1 was developed at the University of Luxembourg and tested by the Centre Hospitalier de Luxembourg, Clinique d'Eich [30, 31].

With this machine, the subject lies in prone position with his knee flexed at 30° to represent the conditions of a Dial test. The foot is positioned within a single-size boot of hard plastic with different internal slippers used to match the individual foot size. With the thigh immobilised to the base, a torque is applied manually to the lower limbs by means of a handle and the produced angle is recorded together with the applied torque. In addition to clinical trials, P1 has been improved by eliminating backlash in the mechanical construction and the boot to measure more objectively the tibia-femoral rotational angle.

## 2. Methods

This paper will now focus on the transition from prototype P1 to prototype P2 in a process of constructive improvements. The previous results on P1 are shortly repeated and then the new features of P2 are presented. Even if P1 was introduced previously [32], a brief description of the first version of the Rotameter is given here below as a reminder.

A torque is applied manually to the lower limb with the handle fixed to the plastic boot. During twisting, the rotational angle is continuously measured by the inclinometer fixed to the handle (NG4U, SEIKA Company, Germany, measuring range of ±80° and resolution of <0.01°). Furthermore, the manually applied torque is measured by strain gauges mounted on the shaft made of a hollow aluminum tube. This shaft is mounted in two roller bearings and ends up with the support-plate fixed at the boot. Torque and rotational angle are simultaneously sampled by a 24-bit A/D converter (NI 9219, National Instruments) and registered by the LabVIEW software from National Instruments. Thanks to the measurements, a two-dimensional torque-angle graph is plotted to illustrate the rotational laxity of the patient's knee versus the external loading.

**Figure 2:**
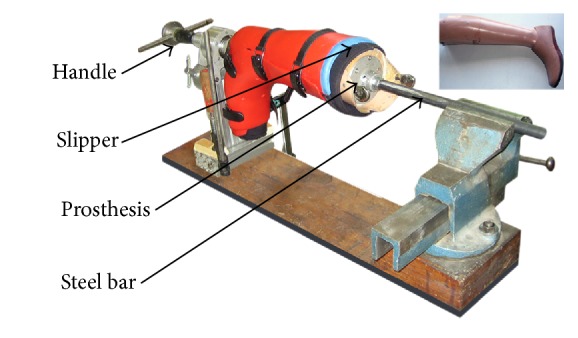
Test on the prosthesis with P1.

A series of trials was conducted with P1 on five subjects with healthy knees and with a lower limb prosthesis of size 40 with a steel bar of 20 mm diameter and 500 mm length attached to its extremity (Figure 2). These subjects were three men and two women between 21 and 31 years with a mean age of 25 years. Their mean height was 174 cm and their mean weight was 75 kg. Due to the one size plastic boot, different slippers had to be used to fill the gap between feet and boot in order to provide a snug fit for the subjects and the prosthesis. Slippers were medium-sized Vacoped (provided by the firm OPED GmbH, Germany), and inflatable and noninflatable slippers ranging from small to extra-large (supplied by ÖSSUR, Iceland).

For the trials with the prosthesis, the slipper was first placed over the artificial limb and then tightened by its Velcro strips. Both the prosthesis and the slipper were then inserted into the boot. For inflatable slippers, air was added inside by continuously pressing the push button. When pressure was reached, the boot was closed by its four straps. Once the prosthesis-slipper-boot assembly process was completed, it was attached to the device by a pipe fixed to the base of the single-size boot and to the clamp by a steel bar (Figure 2). Then, a torque was applied. To change the slipper, the prosthesis was removed from the boot and the new one was fitted in the same way.

When testing healthy subjects with P1, slipper and boot were put on in the same manner as for the prosthesis. The thigh of the volunteer was fixed by splints to avoid movements of the hip joint. During trials, no adverse effects and no skin irritation were noticed by subjects, whatever the torque applied was.

**Figure 3:**
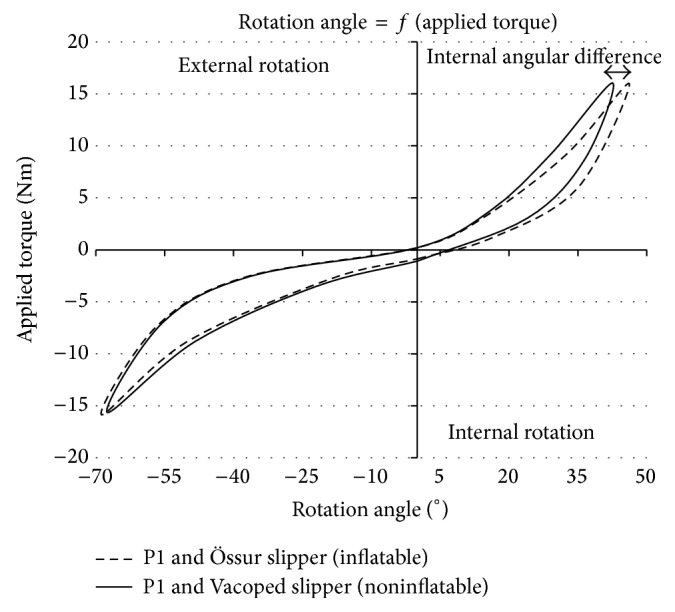
Össur and Vacoped slippers used on prosthesis during twisting with P1.

Results obtained during tests with the prosthesis with different slippers showed an inconvenience of inflatable Össur slippers (Figure 3).

For the external rotation (ER), the maximum rotational angle was slightly more important for the inflatable slipper. Considering the internal rotation (IR), the noninflatable slipper provided a 4° lower maximum angle. The presence of air seemed to alter the exactitude of the measurements by inducing instability within the boot.

**Figure 4:**
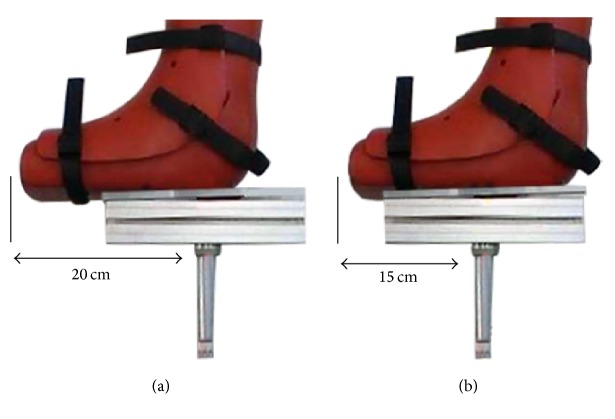
Alignment of the pipe with the (a) anatomical axis and (b) front side of the leg.

Moreover, to test the influence of the position of the pipe on measurements, the hollow tube was fixed at two different places, respectively, around 20 cm and 15 cm from the extremity of the boot. The first position aligns with the anatomical axis of tibia (Figure 4(a)), whereas the second one aligns with the front side of the leg (Figure 4(b)). This 5 cm shift was simply done to check the sensitivity of the measurement with respect to the position of the rotational axis.

**Figure 5:**
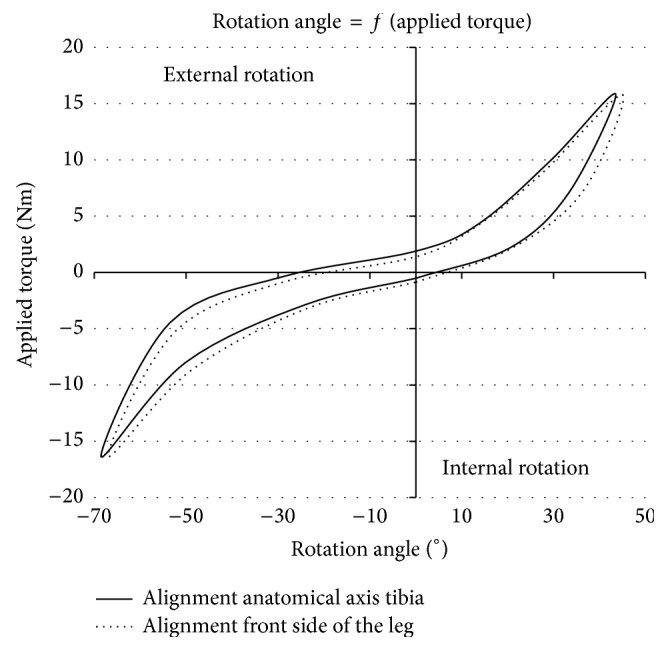
Influence of pipe's alignment.

Figure 5 shows the result obtained on the same volunteer with those two configurations to highlight a possible influence of this alignment.

The curves in Figure 5 illustrate that the measured rotation was not influenced by the alignment. The little variation of maximum 2° of the total rotational laxity measured was negligible compared to other errors.

Furthermore, it was possible to observe the consequence of rotational speed on the hysteresis. Low speed trials showed curves with low energy loss, and higher speed trials provided higher hysteresis surfaces. Therefore, two different acoustic signals have been chosen and rang when either the applied torque or the rotational speed exceeded a predefined limit (e.g., 15 Nm and 0.5 rad/s, resp.).

**Figure 6:**
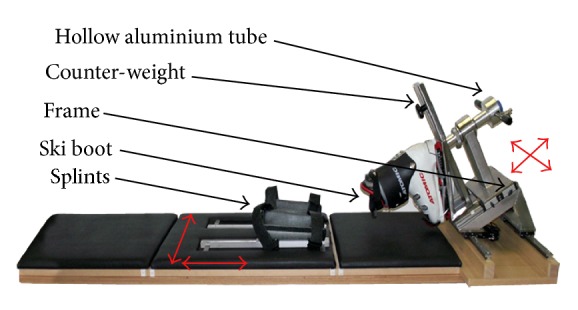
Rotameter P2 and its kinematics.

Despite these results, some problems were detected with the fixation of the thigh highlighted by hip joint mobility. Additionally, the single-size boot and its different types of slippers proved to influence the repeatability of the Rotameter. For example, a trial on a woman with small feet who felt the backlash in the shoe during the test provided unusable results. All of these drawbacks were taken into account when developing the new and improved version P2 of the Rotameter illustrated in Figure 6.

For positionning of the patient's thighs, two cladded cone-shaped half pipes made of stainless steel were mounted on adjustable splints, fixable at any position and completely free of backlash. P2 was also equipped with two pairs of rails that enable its movements lengthwise and across the width to match the size and the anatomy of the patient. Beside this primary function, they also serve to set the zero or the initial position prior to trials. Concerning the frame, it was changed from a screwed aluminium construction (Figure 1) to a welded stainless steel frame (Figure 6) to increase global stiffness. It was mounted in fixable splints free of backlash to adjust it quickly to the individual patient's size. The handle was permanently fixed to the hollow shaft with strain gauges to measure the torque and also welded on a shoe support plate. Thus, the complete shaft with all attachements was a one-piece design which was mounted in two roller-bearings. To match adequately with the patient's foot, a standard ski-boot was fixed to the shoe support plate by an ordinary ski-binding.

Unlike P1 and other devices [23, 24, 33] that are equipped with noninterchangeable boots, P2 offers a variety of sizes by the use of ski shoes. Those boots reduce the ankle's backlash by securing firmly the patient's foot and therefore prevent its mobility within the boot. This system provides a higher rigidity, a good comfort, and practicality as the subject only puts on the boot of his individual size and then steps into the ski-binding. An adjustable counter-weight was installed to balance the different sized ski-boots together with the support, before the patient puts on the boot.

## 3. Results

**Figure 7:**
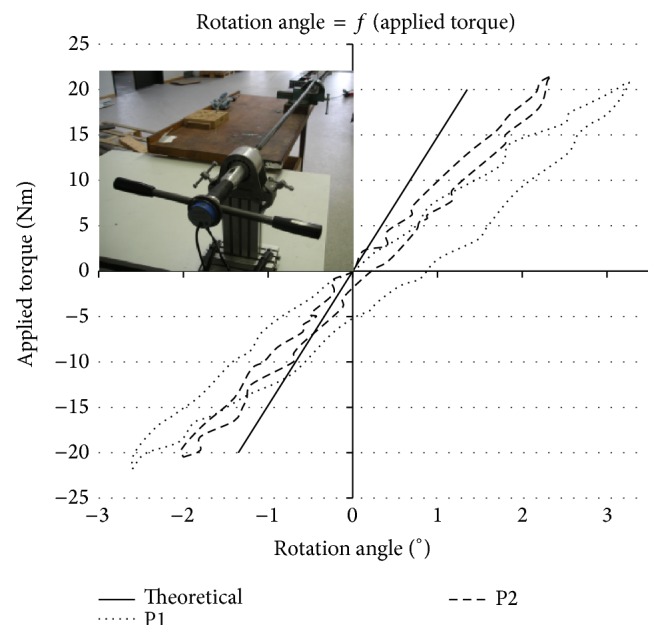
Test set-up with P1, P2 and the theoretical solution.

First tests with a circular steel rod of known length (*L* = 1 500 mm), diameter (*D* = 20 mm), and shear modulus (*G* = 81 000 MPa) were carried out to verifiy the system up to a maximum torque moment of ±20 Nm and to estimate the measurement error inherent to the apparatus (Figure 7). Tests were carried out on P1 and P2 to compare the results. An analitycal solution for the steel rod stiffness was calculated according to the strength of materials in torsion:

(1) $$\begin{array}{c} M_{t}=G\times \frac{\alpha }{L}\times I_{0}, \end{array}$$

where *M*_*t*_: applied torque (Nmm); *G*: shear modulus (MPa); *α*: rotational angle (rad); *L*: length of the steel bar (mm); *I*_0_: polar moment of inertia of the rod (mm^4^).

This led to the following equation for a defined torque and rotational angle:

(2) $$\begin{array}{c} \alpha =\frac{32\times L\times M_{t}}{G\times \pi \times D^{4}}. \end{array}$$

The results obtained with the steel bar for P1 and P2 are visible in the graph in Figure 7 with the analitycal solution calculated up to *M*_*t*_ = ±20 Nm.

The registered angle was very small due to the high stiffness of the steel bar. Nevertheless, when comparing P1 with P2 (dotted and dashed lines, resp.), it is possible to see that the new prototype reduced the measurement error by 1° for both external and internal rotations. A difference of 1° is visible between P2 and the theoretical straight line. The reason for this final error might be an imperfect fixation of the clamped end of the rod on a nonperfect rigid table.

**Figure 8:**
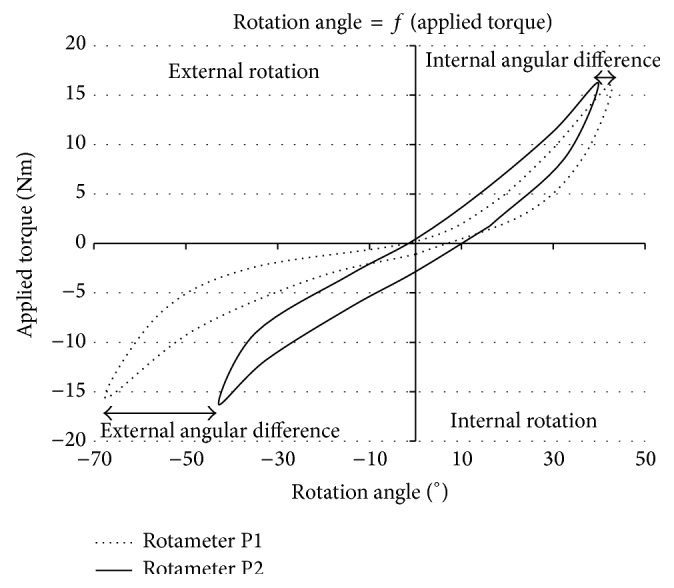
Comparison of P1 and P2 prototypes on the same male test person.

To place the apparatus into work situation and test this improved version, a series of trials was carried out under identical conditions with the same healthy male volunteer on both prototypes P1 and P2.  Figure 8 illustrates the relevance of P2 by showing the variation with respect to prototype P1 equipped with the best slipper (noninflatable Vacoped).

Internal rotation was reduced by 7°, whereas external rotation decreased significantly by 26° showing the positive results of the modifications.

**Figure 9:**
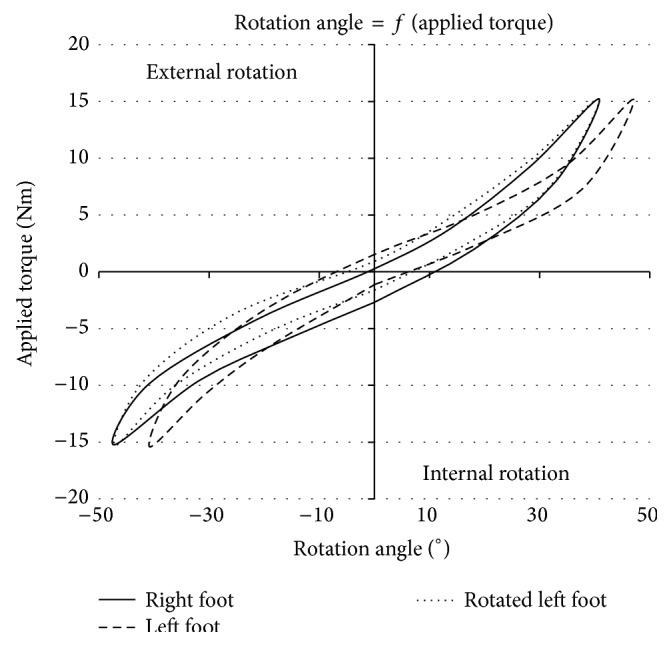
Comparison of right foot, left foot, and rotated left foot of the same healthy patient.

Since our main objective is to develop a device which can measure efficiently TFR and evaluate the treatment after a rupture or a torn ligament, trials were also carried out on P2 to compare left and right rotational knee laxities as shown in Figure 9.

The solid line shows the right foot and the dashed line the left foot as measured. The dotted line represents the left foot curve mirror inverted with respect to the origin of the coordinate system. It is really close to the evolution of the solid line of the right foot. This similarity reflects the possibility to assess the laxity of a human knee after a surgical intervention on a ligament. The comparison between reconstructed and healthy knees allows us to evaluate the effectiveness of a surgical procedure.

**Figure 10:**
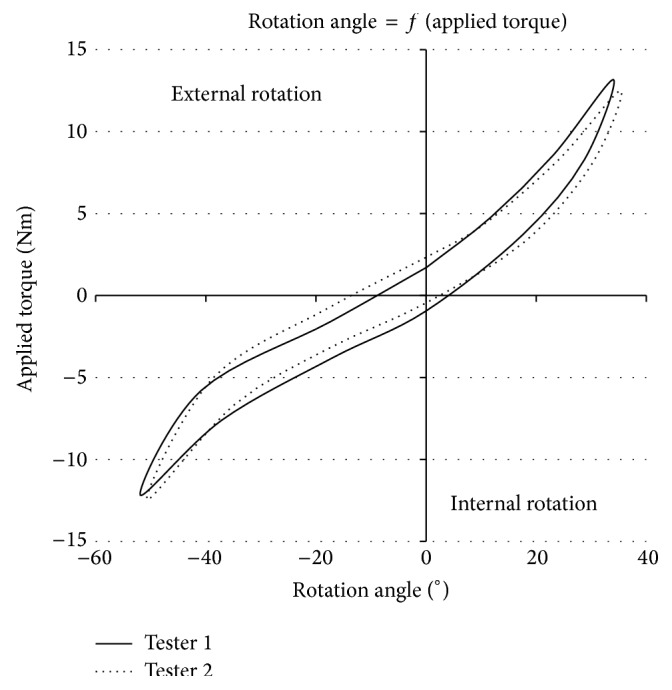
Intertester reliability.

**Figure 11:**
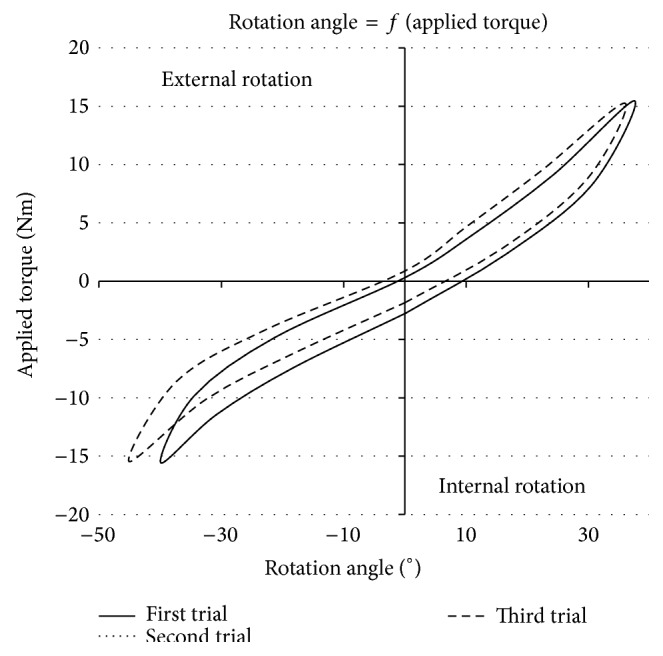
Intratester reliability.

Following the significant reduction of the angular value measured with P2 and in order to evaluate its repeatability, intertester and intratester reliability trials were performed on two healthy subjects, for example, a volunteer for each test. Results are presented in Figures 10 and 11, respectively.

In Figure 10, two different testers measured rotational knee laxity of the same patient's healthy knee under identical operating conditions. The two curves are relatively close to each other and have the same characteristics. Small variations inferior to 3° can be observed for IR and ER.

Another campaign aimed to evaluate the intratester reliability of P2. With that purpose, one single tester performed the trials on the same test person under identical operating conditions. The only difference was the date when those measurements were carried out. Three different moments were considered: Trial 1 and Trial 2 were realised the same day, whereas Trial 3 was carried out four months later. The results of this reliability campaign are shown in Figure 11, one curve for each trial.

First and second trials, in solid and dotted lines, respectively, were carried out on the same day. It was possible to see superimposed curves, showing the repeatability of P2 when used by a tester within one single day. A small variation was observed with third trial in dashed line. This last trial performed four months later was slightly shifted 1° to the left and showed a 6° larger amplitude for the ER. Hence, the error of repeatability can be assessed and is limited to 6° at ±15 Nm torque.

## 4. Discussion

Without any doubt, thickness and material of the slipper influenced the rotational angle on P1. To verify this hypothesis and highlight the varying values resulting from the different slippers, tests were carried out on P1 with an artificial lower limb (Figure 3). Here, the noninflatable Vacoped slipper achieved the best results. An increase of the measured angle was observed when using inflatable Össur slippers of the same size. In this case, increases of 2% and 9% were detected for the maximum ER and IR. The air reduced the stiffness, which was also noticed by Tsai et al. [24] who state that: “*Pressure in the air cells was found to significantly influence the results of the trials*.”

As a consequence, the transition of P1 to P2 was attained by using a ski boot to reduce ankle motion as much as possible. Additionally, the thighs were fixed by means of two rigid metal splints, and a stiffer welded frame was designed.  Figure 8, which considers the same male volunteer tested on both prototypes P1 and P2, proves that the device was improved consequently by these modifications for both rotations, but rather more for the ER. Actually, it was reduced by 55%, 60%, and 38%, respectively, for the torques of −5 Nm, −10 Nm, and −15 Nm. IR also decreased consequently, but this percentage was reduced as the amount of torque: 27%, 14%, and 8% for +5 Nm, +10 Nm, and +15 Nm. Considering the total angular range of male test person up to ±15 Nm, it changed from 110° for P1 (66° in ER and 44° in IR) to 80° for P2 (41° in ER and 31° in IR), which was a considerable decrease of 27%. Those new values illustrated the benefits of a stiffer frame, ski boots, and the use of more rigid splints. The angular range of 80° of the male volunteer found in this study with P2 at ±15 Nm was lower than the one obtained by Lorbach et al. [30] with P1 on 30 healthy volunteers (15 men and 15 women). Authors reached with the first prototype P1 at ±15 Nm an angular range of 119°, which was higher than P2 values. The study of Mouton et al. [34] also confirmed those findings: “*The second prototype of the Rotameter used in the present study yielded lower rotational laxity than the previous one*.”

**Figure 12:**
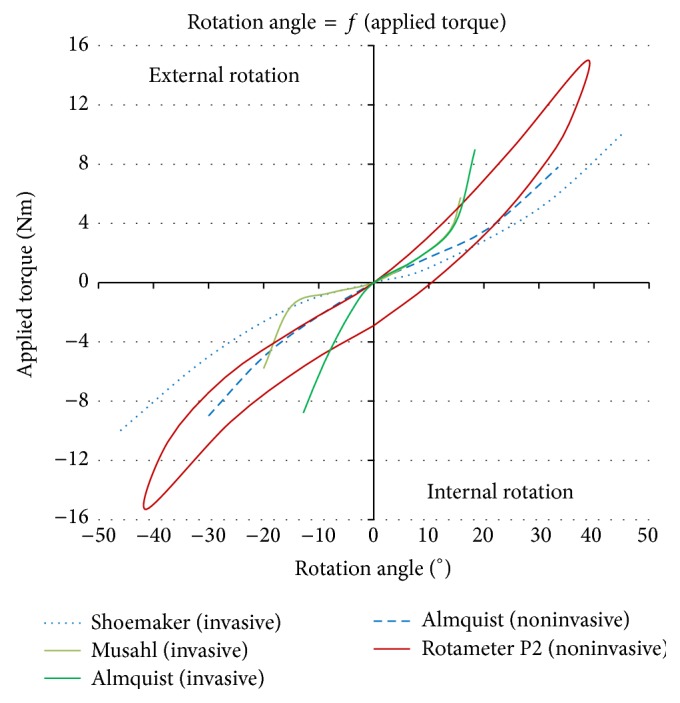
P2 compared to literature data.

Numerous healthy subjects were tested with the first prototype in previous studies [30, 31]. A comparison of this initial model and an invasive navigation system performed on 20 cadaveric knees stripped of all muscles showed high correlations: 0.88 at ±5 Nm, 0.85 at ±10 Nm, and 0.86 at ±15 Nm [30, 31]. In order to ensure the same observation with P2, the intertester and intratester reliabilities were verified. Curves in Figures 11 and 12 confirm these reliabilities to be lower than 6° at torques less than ±15 Nm though subjects were a single volunteer for each test.

### 4.1. Comparison between the New Prototype P2 and Other Noninvasive Devices

During the last decade, several authors developed noninvasive devices to measure tibiofemoral rotation. On a specially designed chair where knee flexion could be adjusted from 0° to 90°, Almquist et al. [33] measured an angular laxity of 65 ± 7° and 66 ± 15° for a ±9 Nm torque at 60° and 90° of flexion, respectively. Shoemaker and Markolf [28] analysed the passive rotation of the knee at 20° and 90° of flexion. Their results showed the impact of the position of the hip on the measured tibia-femoral angle. At 20° and 90° of knee flexion with extended hip, they evaluated 82 ± 19° and 91 ± 17° rotations for ±10 Nm, respectively. Zarins et al. [29] measured the knee rotation on a patient lying sideways. At 30°, 60°, and 90° of flexion, they recorded a total angular laxity of 72°, 73°, and 74°, respectively, but did not mention the torque applied. Almquist et al. [33] and Zarins et al. [29] showed that between 30° and 90° of flexion, the angles of rotation measured with a noninvasive external device exhibited little variation. Indeed, Zarins et al. [29] stated in their paper: “*In accordance with the earlier studies, the range of total rotation did not change significantly between 30*°* and 90*°* of flexion*.” This nonvariation of the angular laxity according to the flexion of the knee allowed comparing P2 working only at 30° of flexion with other noninvasive devices.

A parallelism of previous authors' values with data from the second prototype applied to men showed that P2 measurements are lower. As a matter of fact, Almquist et al. [33], Zarins et al. [29], and Shoemaker and Markolf [28] measured 65 ± 7°, 72°, and 82 ± 19°, respectively, whereas P2 reached 58 ± 3° for the same ±10 Nm torque. Thus, it was possible to notice without any doubts the lower values of P2.

### 4.2. Comparing the New Prototype P2 with Invasive Devices and Medical Imaging Assisted Devices

Invasive computer-assisted studies also enable the measurement of rotational knee laxity. In the course of their work, Almquist et al. [33] measured the rotational laxity range of 5 male subjects at 60° and 90° of flexion by Roentgen Stereometric Analysis (RSA) with markers placed on the proximal tibia and the distal parts of the femur. They recorded rotations of 22 ± 6° and 25 ± 7° for a ±6 Nm torque. In their study, Musahl et al. [23] focused on cadavers at 30° of flexion with ±6 Nm. They measured a total rotation of 42° thanks to electromagnetic sensors attached to the bones. With this very same device, Tsai et al. [24] obtained noninvasively a total rotational laxity of 26 ± 6° at 30° of flexion with a 6 Nm torque by use of magnetic trackers mounted on the boot, tibia, and femur. Hemmerich et al. [35] used Magnetic Resonance Imaging (MRI) to measure the rotation of the knee by combining this medical imaging apparatus and their prototype. The patient supine with the knee flexed at 30° provided a 28° total rotation for 5,8 Nm applied.

As we can see in Figure 12, when applying the same torques and then comparing the rotational angles measured by these various devices with the new prototype P2, the results achieved with P2 are very similar. Musahl et al. [23] obtained invasively a total rotation of 42° at ±6 Nm whereas the new prototype P2 yielded approximately 37° at ±5 Nm. But it should be considered that the mechanical properties of cadaveric knees may have altered by postmortem effects. In fact, Tsai et al. [24] used the same device on living volunteers and obtained noninvasively lower angular values, despite the artefacts resulting from the fixation of trackers on the skin and the difficulty of operating this technology. Almquist et al. [33] obtained 22 ± 6° and 25 ± 7° at ±6 Nm torque for a flexion of 60° and 90° of the knee. The same order of magnitude, 28° of total laxity, was obtained by Hemmerich et al. [35] with MRI technique. Those last values, about 10° lower than P2, were reached by the use of RSA and MRI that allow measurements without any movements of soft tissues and adjacent joints. These medical imaging techniques provided smaller rotational angles compared to P2, but their process exhibited the patient to radiation issues as, for example, in scanners and other devices. MRI could appear suitable for measuring TFR, though it is more time consuming and expensive to operate.

### 4.3. Comparing Right and Left Knees

The rotational comparison between right and left knees has also been an interesting topic for many researchers. Indeed, measuring the rotational laxity of an operated knee and a healthy one could help evaluating the return to the initial laxity. Moreover, the efficiency of surgical procedures aiming at restoring rotational laxity could be evaluated directly by comparing the characteristic curves of the two knees.

In the study of Tsai et al. [24] with 11 volunteers, mean rotational laxities were 27° and 28° for left and right knees, respectively. The difference between both sides was therefore slightly more than 1° with a ±6 Nm torque. Shoemaker and Markolf [28] stipulate the same observation: “*The mean rotation and standard deviations were nearly identical for right and left knees, demonstrating that for our sample population the difference between the means of the right and the left sides was approximately zero*”. This phenomenon was also observed by Mouton et al. [34] with P2: “*No significant differences were found between the left and the right knee of healthy subjects*.” This observation could now appear helpful to evaluate surgical interventions. Furthermore, P2 could also be used to evaluate sports where left and right thighs and knees are differently activated and charged. Additionally, it could compare rotational knee laxity between high-level athletes and less active population to investigate possible variations.

## 5. Conclusion

The second Rotameter prototype P2 contributed to a significant reduction in the measured tibia-femoral rotational angle compared to P1, and the repeatability was reduced to 6° at ±15 Nm torque. The present study allowed demonstrating that the left and right rotational knee laxities of healthy subjects are almost identical. Hence, the comparison between abnormal knee and the healthy one could be useful for clinical purposes. Prototype P2 may be a suitable device to measure noninvasively in an easy way tibia-femoral rotation, to observe rotational stability, and to evaluate surgical reconstruction. In particular, as the device is portable, simple, and user-friendly, P2 is currently in use in clinical studies to set up a database for healthy volunteers and injured patients and to assess the repeatability on a large number of subjects.

A forthcoming study with Rotameter P2 will evaluate invasively its absolute precision.
